# RYK promotes the stemness of glioblastoma cells via the WNT/β-catenin pathway

**DOI:** 10.18632/oncotarget.14564

**Published:** 2017-01-09

**Authors:** Assunta Adamo, Danilo Fiore, Fabio De Martino, Giuseppina Roscigno, Alessandra Affinito, Elvira Donnarumma, Ilaria Puoti, Lucia Ricci Vitiani, Roberto Pallini, Cristina Quintavalle, Gerolama Condorelli

**Affiliations:** ^1^ Department of Molecular Medicine and Medical Biotechnology, “Federico II” University of Naples, Naples, Italy; ^2^ IEOS, CNR, Naples, Italy; ^3^ IRCCS-SDN, Naples, Italy; ^4^ Department of Hematology, Oncology and Molecular Medicine, Istituto Superiore di Sanità, Rome, Italy; ^5^ Institute of Neurosurgery, Università Cattolica del Sacro Cuore, Rome, Italy

**Keywords:** glioblastoma, stem cells, ryk, β-catenin

## Abstract

Glioblastoma multiforme (GBM) is characterized by a strong self-renewal potential and a poor differentiation state. Since receptor-like tyrosine kinase (RYK) activates the WNT/β-catenin pathway essential for cancer stem cell maintenance, we evaluated its contribution in conferring stemness to GBM cells. Here, we report that Ryk (related-to-receptor tyrosine kinase), an atypical tyrosine kinase receptor, is upregulated in samples from GBM patients as well as in GSCs. Ryk overexpression confers stemness properties to GBM cells through the modulation of the canonical Wnt signaling and by promoting the activation of pluripotency-related transcription factor circuitry and neurosphere formation ability. In contrast, siRNA-mediated knockdown of Ryk expression suppresses this stem-like phenotype. Rescue experiments reveal that stemness-promoting activity of Ryk is attributable, at least in part, to β-catenin stabilization. Furthermore, Ryk overexpression improves cell motility and anchorage independent cell growth. Taken together, our findings demonstrate that Ryk promotes stem cell-like and tumorigenic features to glioma cells its essential for the maintenance of GSCs and could be a target of novel therapies.

## INTRODUCTION

Glioblastoma multiforme (GBM) is the most common and aggressive primary brain tumor and one of the most devastating human malignancies. Recent studies have suggested that this tumor arises from a small population of cancer stem cells (CSCs, or tumor-initiating cells). CSCs retain many properties of normal neural stem cells (NSCs), such as self-renewal and multipotency [1, 2]. They are believed to constitute the tumor's driving force and to be responsible for tumor recurrence and radio- and chemo-resistance [3–6]. Indeed, blocking self-renewal signaling by targeting surface markers or forcing differentiation of GBM CSCs (GSCs) may represent potential effective therapeutic strategies for GBM. However, poor understanding of the molecular pathways involved in CSCs expansion and maintenance has hindered the development of such approaches.

The WNT/β-catenin signaling pathway is essential in regulating self-renewal and proliferation or differentiation of NSCs and CSCs. After binding cell-surface receptors, WNT initiates a transduction cascade that stabilizes the transcriptional co-activator β-catenin, allowing it to translocate to the nucleus, form a transcriptional activation complex with TCF/LEFT (T-cell specific transcription factor/lymphoid enhancer-binding factor), and initiate transcription of targeted genes [7]. Deregulation of WNT signaling in NSCs or progenitor cells due to genetic or epigenetic alterations can promote CSCs survival [8]. On the other hand, persistent β-catenin activation has also been demonstrated in GSCs, promoting self-renewal, invasiveness, neurosphere formation, and epithelial-to-mesenchymal transition (EMT) [9, 10].

Receptor-like tyrosine kinase, or related-to-receptor tyrosine kinase (RYK), is an atypical member of the receptor tyrosine kinase (RTK) family. It is characterized by an impaired kinase activity [11] and an extracellular WNT inhibitory factor domain that binds WNT ligands, leading to the activation of β-catenin-dependent pathways [12, 13]. RYK controls fundamental biological processes, including neuronal differentiation and axon outgrowth [13, 14], and its deregulation has been proven to be associated with cancer: indeed, RYK is overexpressed in ovarian cancer tissues [15], is essential for WNT-5a-dependent invasiveness in glioma, and its expression correlates with the WHO's histological grading system for glioma tissues [16].

Here, we describe a hitherto unknown function of RYK in establishing the stem-like phenotype of GBM cells. We provide evidence that the receptor plays a key role in conferring stemness to GBM cells by regulating β-catenin expression and function. RYK may therefore represent a new target for the treatment of GBM.

## RESULTS

### RYK is overexpressed in glioblastoma and stem-like glioblastoma cells

**Figure 1:**
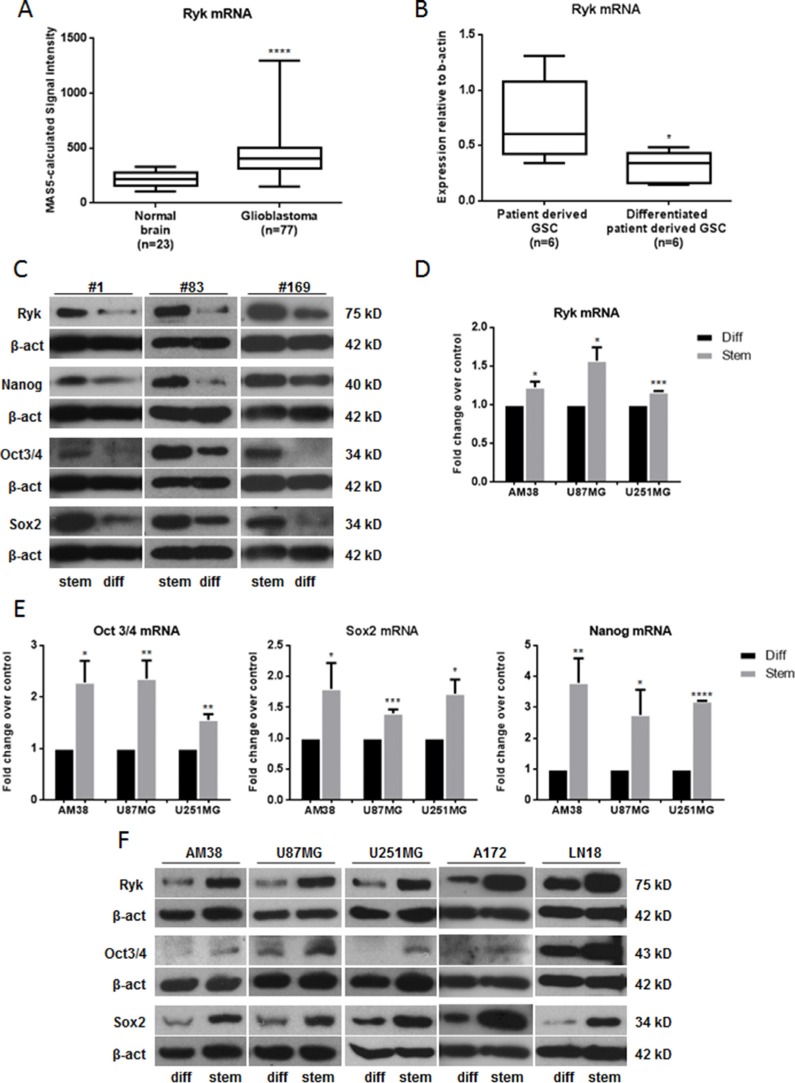
RYK is overexpressed in GBMs and GSCs (**A**) A significant increase in RYK expression was identified in GBM tissues (*n* = 77) compared to normal brains (*n* = 23). RYK expression data was obtained from the GEO Profiles database. (**B**) RYK's mRNA expression is greater in patient-derived GSCs (*n* = 6) compared to patient-derived GSCs induced to differentiate (*n* = 6). RYK expression was assessed by real-time PCR and normalized against β-actin. In (**C**), RYK, NANOG, SOX2, and OCT3/4 protein levels were assessed by Western Blot in three patient-derived GSCs (#1, #83 and #169) and their differentiated counterpart. Real-time PCR (**D**, **E**) and/or Western blotting (**F**) were performed to analyze RYK, NANOG, SOX2, and OCT3/4 mRNA and/or protein levels in differentiated and stem-like GBM cell lines (AM38, U87MG, U251MG, A172 and LN18). In c and f, Western blots from representative experiments; β-actin was used as loading control. In (C) the experiments were repeated at least twice. In (A, B, D and E), statistical significance calculated using Student's *t*-test (*p* < 0.05 considered significant). Results presented as mean ± SD. *p < 0.05; **p < 0.01; ***p < 0.001; ****p < 0.0001. In (C) the blots representing Ryk, Nanog, Oct3/4 and Sox2 for patient #1 are from the same gel. The blots representing Ryk and Oct3/4 for patient #83 are from the same gel. The blots representing Ryk and Oct 3/4 and the blots representing Nanog and Sox2 for patient #169 are from the same gels. In (F) the blots representing Ryk and Sox2 for AM38 are from the same gel. The blots representing Ryk, Oct 3/4 and Sox2 for A172 are from the same gel. The blots representing Ryk and Oct3/4 for LN18 are from the same gel. Therefore they have the same β-actin normalization.

We gathered RYK expression data on 23 normal brain samples and 77 GBM samples from the Gene Expression Omnibus (GEO) Profiles database [17] (GEO accession number: GDS1962). Expression of RYK mRNA was significantly increased in tumors compared to normal tissue (*p*-value < 0.0001) (Figure 1A), a trend confirmed by data obtained from the Oncomine database [18](Supplementary Figure 1A).

RYK mRNA expression was then analyzed in six patient-derived GSC lines and six patient-derived GSC lines induced to differentiate. As shown in Figure 1B, RYK was expressed at a greater extent in GSCs than in differentiated cells. Furthermore, we showed a decrease of RYK protein levels in three patient derived GSCs compared to their differentiated counterpart (Figure 1C), suggesting a possible role for RYK in the maintenance and promotion of CSCs. Of note, neurospheres derived from different continuous GBM cell lines had high levels of RYK mRNA (Figure 1D) and/or protein (Figure 1F) beside other well-established GSC markers, including NANOG, SOX2, OCT3/4 (Figure 1E and 1F), NESTIN, CD133, and EZH2 (Supplementary Figure 1B and 1C). Taken together, these findings indicate that RYK is strongly upregulated in GBM stem cells and are suggestive of a possible role of this receptor in the promotion of stemness.

### Knockdown of RYK inhibits self-renewal in GSCs

**Figure 2:**
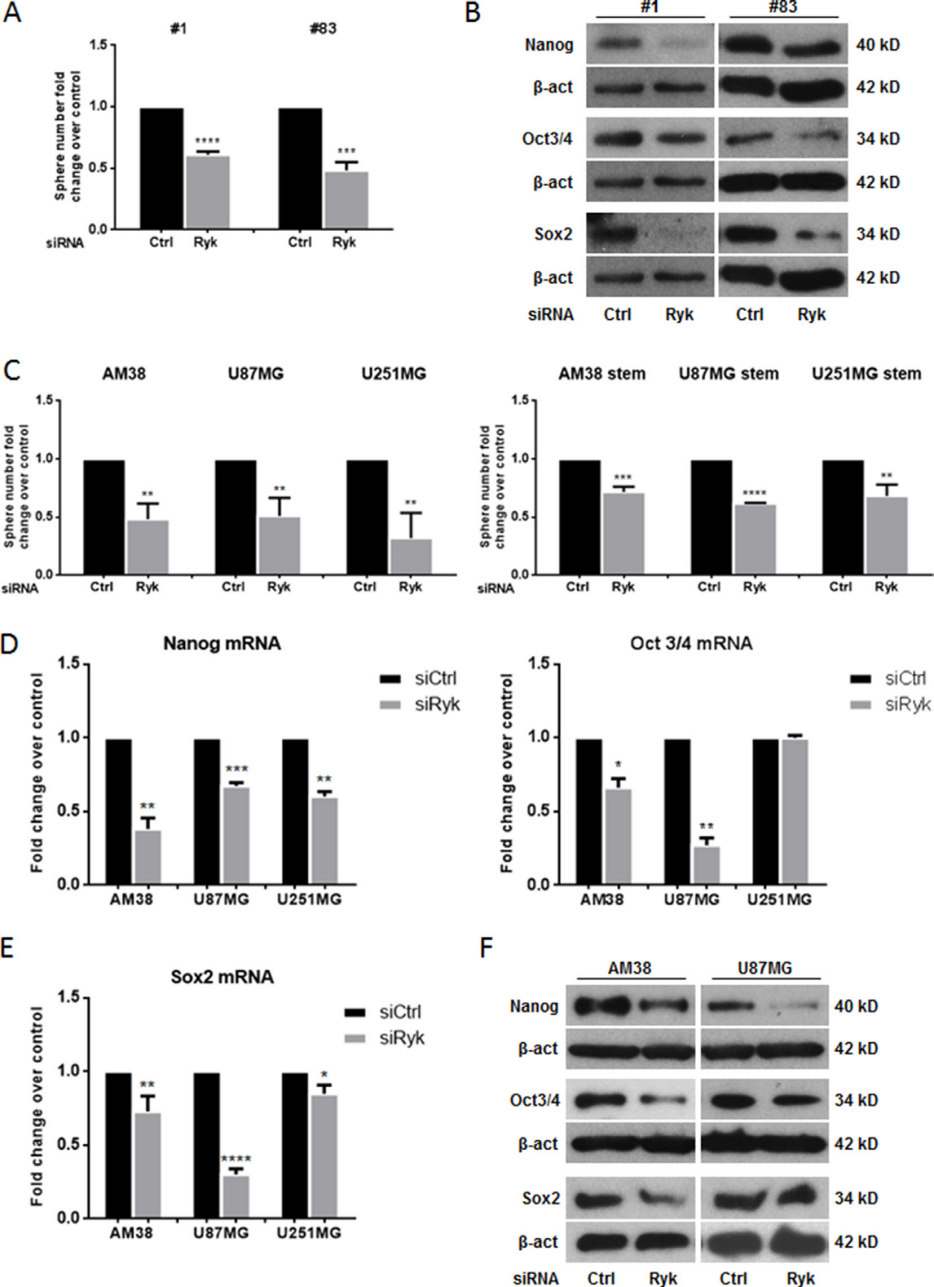
RYK silencing affects neurosphere formation GBM patient-derived stem-like cells (#1, #83) or GBM cell lines (AM38, U87MG, and U251MG) were transfected with Ryk siRNA or a control siRNA sequence. The ability to grow as neurospheres and the expression of stem markers was then analyzed. Knock-down of RYK expression reduced sphere number in both patients analyzed (**A**) as well as in the GBM cell lines (**C**). Data representative of three independent experiments. RYK knockdown also decreased the GSC markers NANOG, OCT3/4, and SOX2 at mRNA and protein levels (**D**–**F**). In (D) and (E) mRNA expression was assessed by real-time PCR and normalized against β-actin. Experiments were repeated at least twice. In (**B**) and (F), Western blots from representative experiments; β-actin was used as loading control. In (A, C, D, and F), statistical significance calculated using Student's *t*-test (*p* < 0.05 considered significant). Results presented as mean ± SD. *p < 0.05; **p < 0.01; ***p < 0.001; ****p < 0.0001. In (B) the blots representing Nanog, Oct3/4 and Sox2 for patient #1 are from the same gel. The blots representing Nanog and Sox2 for patient #83 are from the same gel. Therefore they have the same β-actin normalization.

We next investigated the effect of silencing RYK on two different patient-derived GSC lines (patients #1 and #83) [19]. The ability of GSCs to form neurospheres *in vitro* was drastically impaired by knocking down RYK expression with a specific siRNA (Figure 2A); moreover, GSC markers were decreased (Figure 2B). To further confirm the role of RYK as a stemness promoter in GBM, we transfected siRyk or siRNA control sequences into three continuous GBM cell lines under either adherent or stem cells-enriched conditions. RYK silencing was evaluated by RT-PCR and western blot (Supplementary Figure 2A and 2B). As expected, GBM cells with silenced expression of RYK had a significantly impaired ability to form neurospheres (Figure 2C).

RYK knockdown induced a decrease of the expression of stem markers as assessed by RT-PCR (Figure 2D–2E) and/or Western blotting (Figure 2F and Supplementary Figure 2B).

### RYK overexpression promotes neurosphere formation

**Figure 3:**
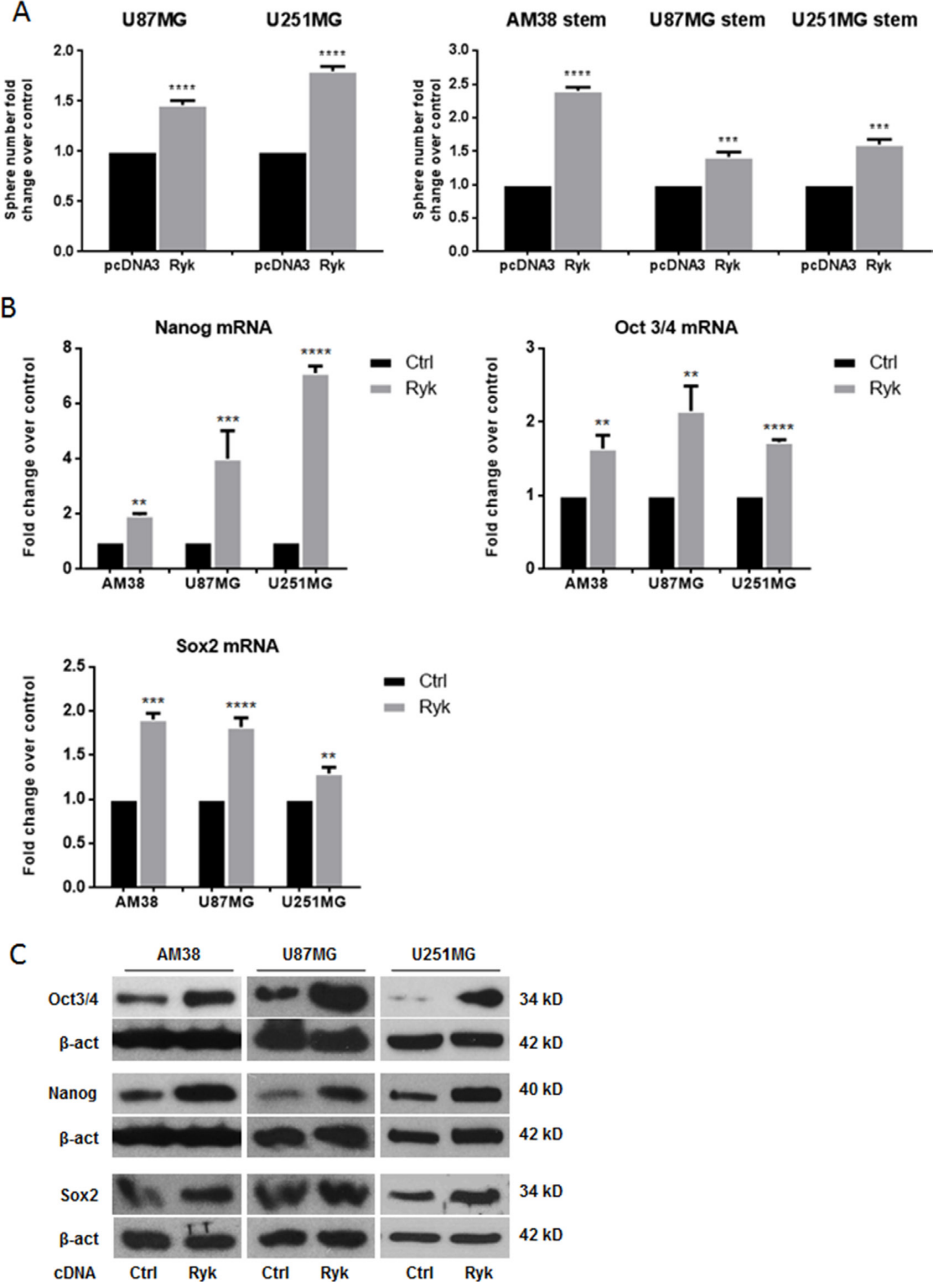
RYK overexpression promotes neurosphere formation Adherent GBM (U87MG and U251MG) and stem-like derived cells (AM38, U87MG, and U251MG) were transfected with h-*RYK* cDNA or a control vector. The ability to grow as neurospheres and the expression of stem markers were then analyzed. RYK overexpression increased sphere number in GBM cell lines (**A**, left panel) as well as in stem-like GBM cells (A, right panel). Data representative of three independent experiments. RYK knockdown also decreased the GSC markers NANOG, OCT3/4, and SOX2 at mRNA and protein levels in all continuous cell lines analyzed (**B**–**C**). In (B), mRNA expression was assessed by real-time PCR and normalized against β-actin. Experiments were repeated at least twice. In (C), Western blots are from representative experiments, and β-actin was used as loading control. In (A) and (B), statistical significance calculated using Student's *t*-test (*p* < 0.05 considered significant). Results presented as mean ± SD. **p < 0.01; ***p < 0.001; ****p < 0.0001. In (C) the blots representing Oct3/4 and Nanog for AM38 are from the same gel. Therefore they have the same β-actin normalization.

To move forward in the understanding of the oncogenic potential of RYK in GSCs and continuous cell lines, we performed a sphere-formation assay in AM38, U87MG and U251MG. We found that the formation of spheres was significantly enhanced when RYK was overexpressed in differentiated and in stem-like cells (Figure 3A). Furthermore, transfection of differentiated GBM cells with h-*RYK* increased stemness markers at mRNA and protein levels (Figure 3B–3C and Supplementary Figure 3A). RYK overexpression was assessed by western blot, as shown in Supplementary Figure 3B. Taken together, these findings confirm that RYK plays a central role in neurosphere formation.

### RYK enhances stem cell frequency, anchorage-independent growth and cell migration

**Figure 4:**
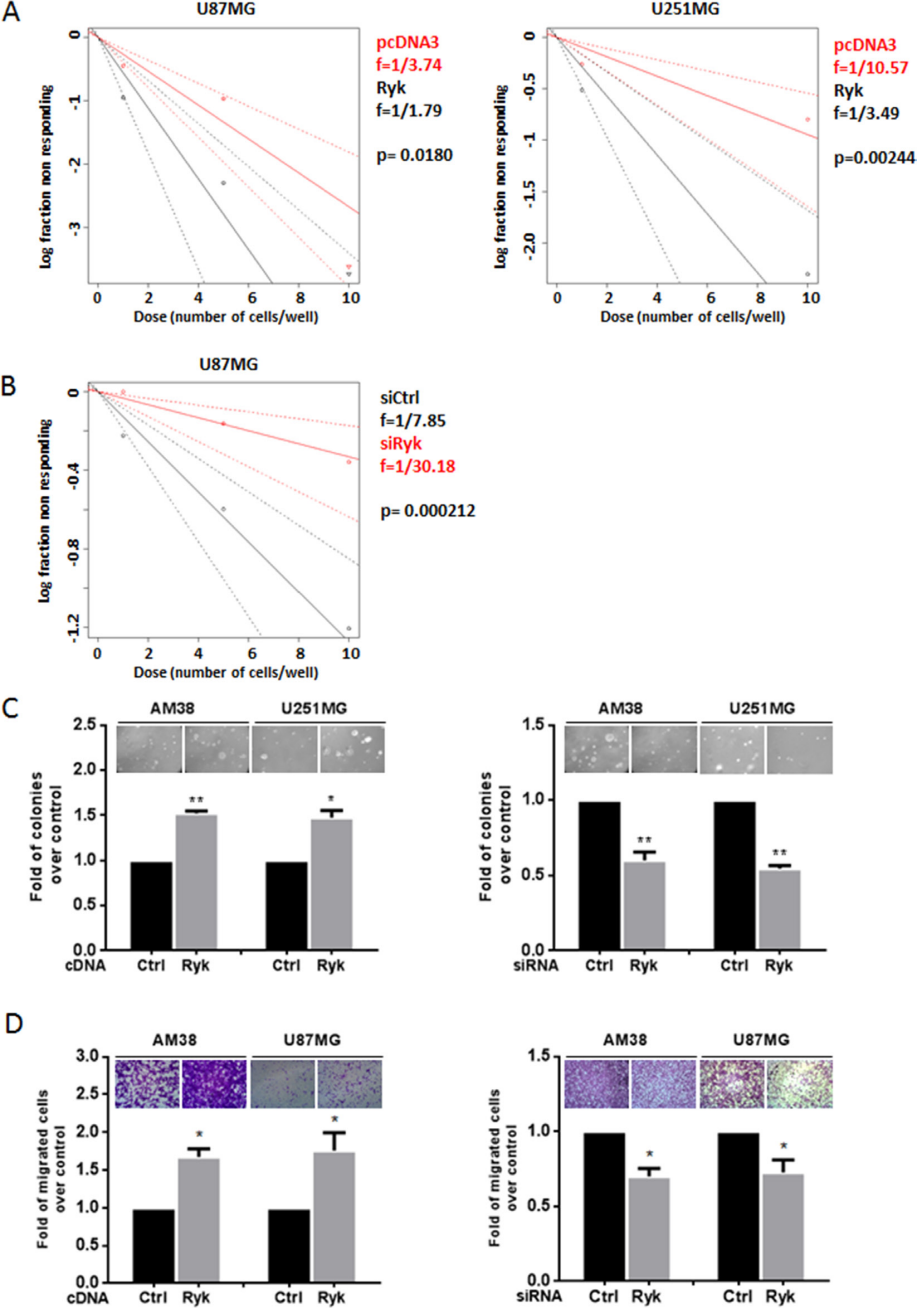
RYK's effects on GBM cells Glioblastoma cell lines (AM38, U87MG, and U251MG) were transfected with h-*RYK* cDNA or with an siRyk-RNA sequence. The *in vitro* LDA revealed that RYK overexpression enriched the stem-like population in U87MG and U251 cell lines (**A**). On the contrary, RYK knockdown in U87MG reduced the stem cells frequency (**B**). Limiting dilution analyses were performed using Extreme Limiting Dilution Analysis (http://bioinf.wehi.edu.au/software/elda). (**C**) Anchorage-independent cell growth was analyzed by a soft-agar assay 14 days after transfection. RYK overexpression promoted anchorage-independent cell growth (left panel), whereas its knockdown produced an opposite effect (right panel). Data representative of two independent experiments. (**D**) Cell migration was analyzed by a transwell migration assay. RYK overexpression increased cell migration (left panel), an effect reverted by silencing (right panel). Data in (C) and (D) representative of three independent experiments. Statistical significance calculated using Student's *t*-test (*p* < 0.05 considered significant). Results presented as mean ± SD. *p < 0.05; **p < 0.01; ***.

We next performed limiting dilution assay (LDA) in U87MG and U251MG cell lines under sphere-forming condition. Data were analyzed using ELDA (Extreme Limiting Dilution Analysis) software [20]. Cells transfected with h-RYK showed an increased spheroid frequency, demonstrating that RYK expression is able to enrich the stem-like population (Figure 4A). Conversely, RYK knockdown resulted in a reduced stem cells frequency (Figure 4B). Moreover, to explore other cancer-promoting effects of RYK, we investigated whether it had an impact on anchorage-independent cell growth and on cell migration. AM38 and U251MG cells forced to overexpress RYK had an increased capability to form colonies in a semi-solid medium (Figure 4C, left panel); in contrast, RYK knockdown determined a reduction in colony formation (Figure 4C, right panel). Moreover, the migration of AM38 and U87MG cell lines was increased upon overexpression of RYK (Figure 4D, left panel), and lost upon knockdown (Figure 4D, right panel). These results clearly demonstrate that, besides behaving as a stemness promoter, RYK acts as an oncogenic factor, enhancing anchorage-independent cell growth and cell migration in GBM.

### β-Catenin is a key molecule of RYK-mediated effects

**Figure 5:**
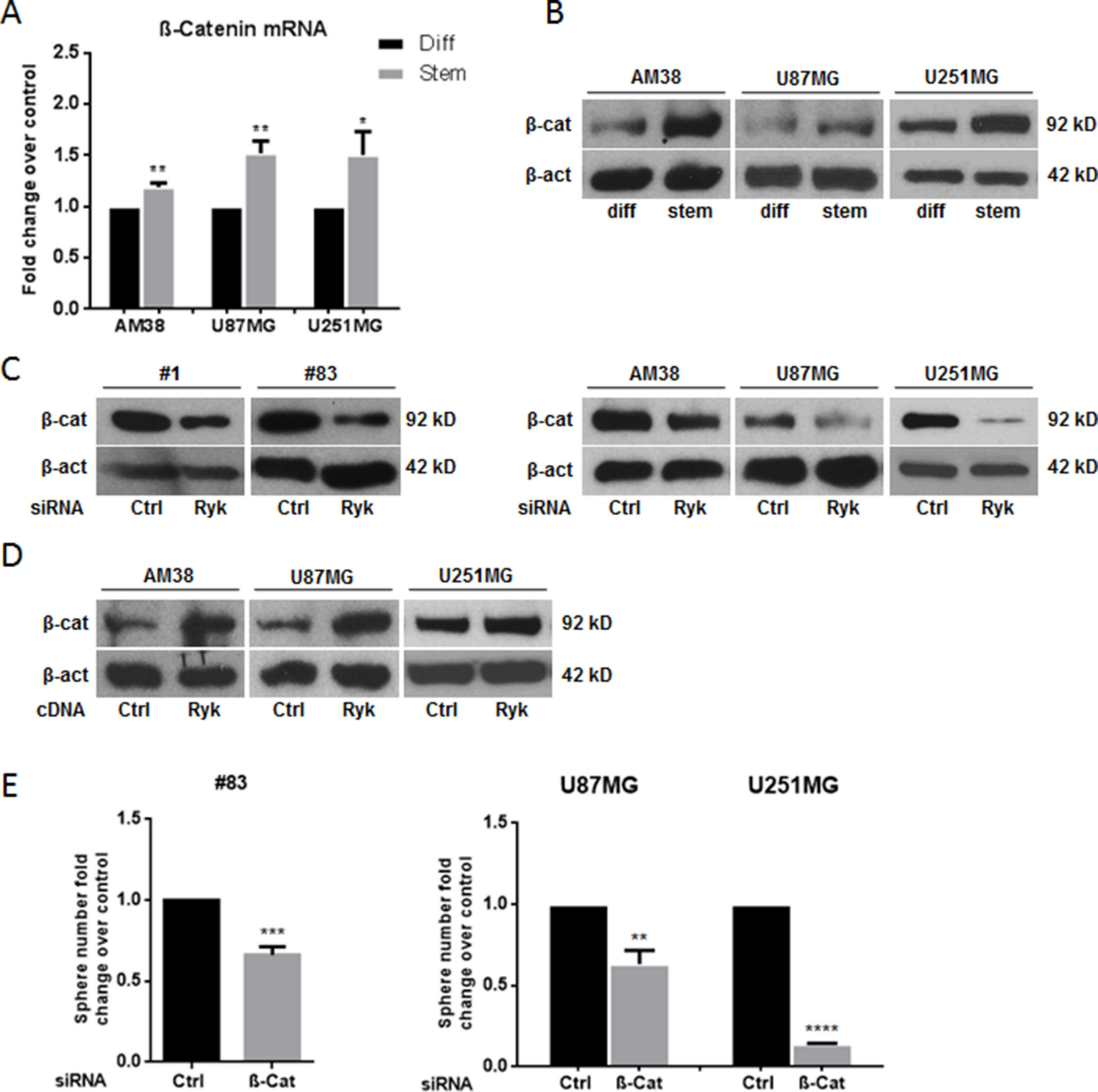
RYK's effects on stem phenotype are mediated by β-catenin β-catenin mRNA and protein levels were assessed in differentiated and in stem-like GBM cell lines (AM38, U87MG, and U251MG) by real-time PCR (**A**) and Western blotting (**B**). Glioblastoma patient-derived stem-like cells (#1 and #83) and differentiated cells (derived from AM38, U87MG, and U251MG cells) were transfected with a *RYK* siRNA sequence and/or *RYK* cDNA. RYK knockdown decreased β-catenin in all the cell lines analyzed (**C**). In contrast, RYK overexpression improved protein level in GBM lines (**D**). GBM patient-derived stem-like cells (#83) and GBM cell lines (AM38 and U251MG) were transfected with a β-catenin siRNA or a control siRNA and the ability to grow as neurospheres was then analyzed. β-Catenin knockdown reduced sphere number in GSCs from patient #83 and in the two GBM cell lines analyzed (**E**). Data representative of three independent experiments. In (A), mRNA expression was assessed by real-time PCR and normalized against β-actin. Experiments were repeated at least twice. In (B), (C), and (D), Western blots from representative experiments; β-actin was used as loading control. In (A) and (E), statistical significance calculated using Student's *t*-test (*p* < 0.05 was considered significant). Results presented as mean ± SD. *p < 0.05; **p < 0.01; ***p < 0.001; ****p < 0.0001. In (B) the blot representing β-catenin for AM38 is from the same gel of Figure 1F. In (C) the blot representing β-catenin for patient #83 is from the same gel of Figure 2B. The blot representing β-catenin for AM38 is from the same gel of Figure 2F. In (D) the blots representing β-catenin for AM38 and U87MG are from the same gel of Figure 3C. Therefore they have the same β-actin normalization.

Since RYK is involved in the WNT/β-catenin pathway, we reasoned that its role as a stemness promoter in GBM may be mediated by stabilization of β-catenin. Indeed, expression of β-catenin mRNA and protein was higher in cell line-derived GSCs compared to their differentiated counterpart (Figure 5A, 5B). Furthermore, transfection of siRyk in GSCs derived from patients #1 and #83 and in GBM cells downregulated β-catenin expression (Figure 5C), whereas overexpression of RYK in GBM cells upregulated β-catenin (Figure 5D). Coherently, knock-down of β-catenin with a specific siRNA mimicked the effects of siRyk transfection, reducing neurosphere formation in patient #83-derived GSCs and in U87MG and U251MG cells (Figure 5E).

### β-catenin overexpression rescues the RYK-mediated stemness phenotype

**Figure 6:**
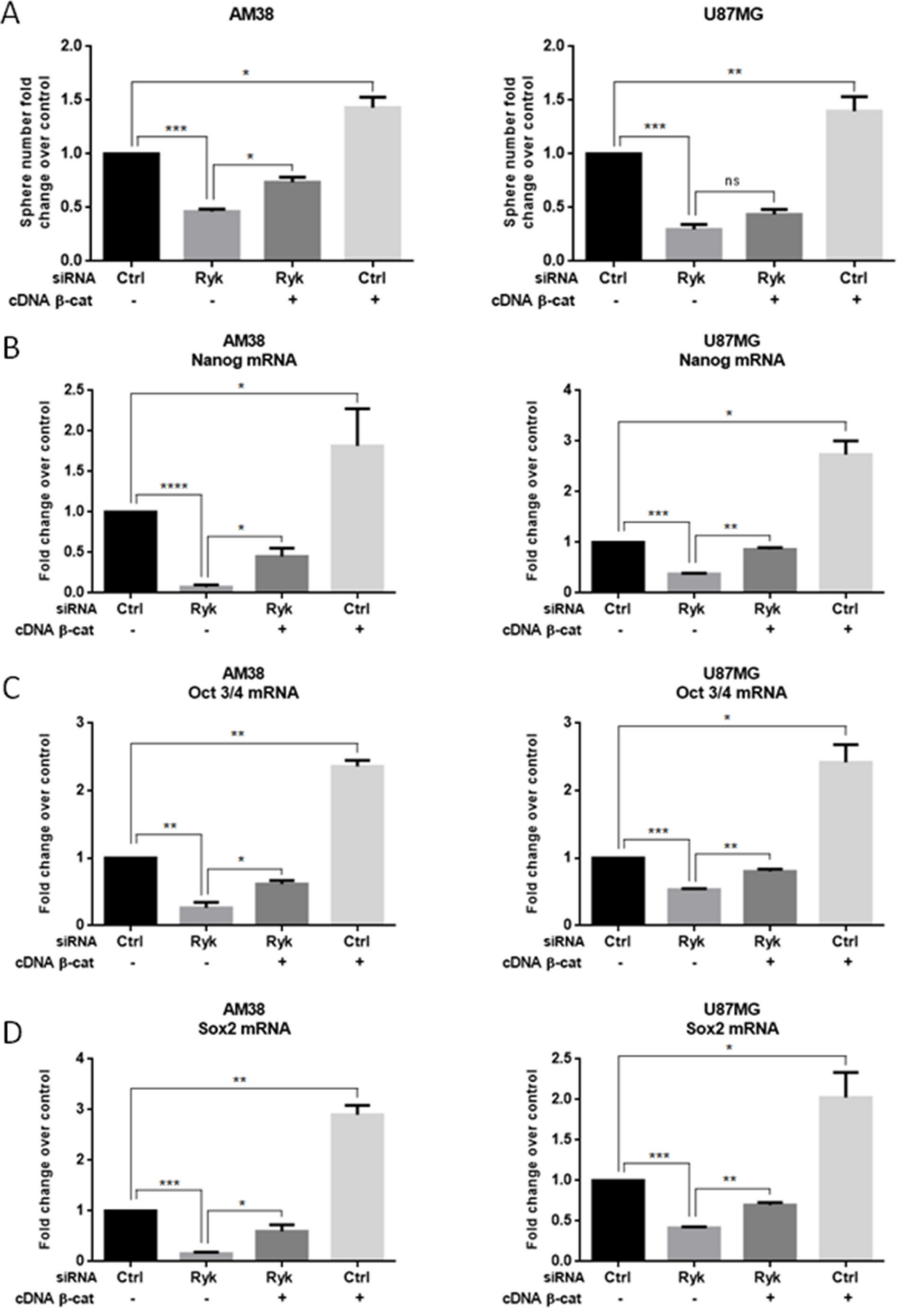
β-Catenin overexpression rescues the effect of RYK knockdown on stemness AM38 and U87MG cells were co-transfected with *RYK* siRNA or a control siRNA sequence, and with either a vector carrying β-catenin or a control sequence. Exogenous β-catenin expression was able to partially counteract the effects of RYK silencing on the ability to form neurospheres (**A**) and on stem marker expression (**B**–**D**). In (A), data representative of three independent experiments. In (B–D), mRNA expression was assessed by real-time PCR and normalized against β-actin. Experiments were repeated at least twice. Statistical significance was calculated using Student's *t*-test (*p* < 0.05 considered significant). Results presented as mean ± SD. *p < 0.05; **p < 0.01; ***p < 0.001; ****p < 0.0001.

To demonstrate a causal link between RYK and β-catenin in determining stemness, we performed a rescue experiment by transfecting GBM cells with siRyk together with β-catenin cDNA. Expression of exogenous β-catenin counteracted the effect of siRyk on neurosphere formation (Figure 6A) and increased the expression of stem-cell markers (Figure 6B–6D). Taken together, these findings strongly support the hypothesis that the role of RYK in promoting the stemness of GBM is mediated, at least in part, by the stabilization of β-catenin.

## DISCUSSION

RYK is an atypical member of the RTK family able to function as a WNT co-receptor and activate the canonical β-catenin-dependent pathway. RYK has been previously linked to ovarian cancer and brain tumors [15, 16], but its role in the pathogenesis of GBM has been poorly investigated. Here we demonstrate for the first time that RYK promotes the stemness properties of GBM cells and is involved in GBM stem cell maintenance. Indeed, we found RYK dramatically upregulated in a large cohort of GBM samples and in GBM cancer stem cells either obtained directly from patients or derived from commercially available GBM cell lines. These results enforce previous observations on the oncogenic role of RYK in GBM [16, 21], and are indicative of a possible key role of the receptor in GSC maintenance.

GSCs are characterized by the ability to grow as neurospheres under appropriate culture conditions and by uncontrolled expression of one or more pluripotency-related transcription factors, such as NANOG, OCT3/4, and SOX2. Minimal expression of these regulatory genes in normal brain cells promotes maintenance of quiescence and low self-renew in the NSC compartment; overexpression of one or more of these factors leads to a GSC phenotype via oncogenic activation, transformation, and aberrant expansion of a mutated cell pool [22]. Our knock-down and forced-expression studies demonstrate that RYK modulates neurosphere formation and the expression of OCT3/4, SOX2, and NANOG in GSCs derived from three cell lines and two patients.

Moreover, we demonstrate that when RYK is overexpressed in differentiated cells, it promotes de-differentiation and conversion toward a stemness phenotype. This suggests that RYK not only sustains the stemness features of GSCs, but also promotes a shift to a more undifferentiated state. Therefore, RYK may exert a tumorigenic role by enriching the GSCs pool or by initiating malignant transformation of cells. Consistent with this, RYK overexpression has been reported to induce the acquisition of tumorigenic properties in the mouse fibroblast cell line NIH3T3 [21].

We also observed that GBM cells are strongly dependent on RYK for anchorage-independent growth and for migration. This is in agreement with results obtained by Habu *et al*. demonstrating that RYK is required for the WNT-5a-dependent invasive activity of glioma cells [16], and suggests that RYK participates in conferring a more aggressive phenotype.

We hypothesized that the observed effects were mainly mediated through the regulation of β-catenin expression levels. Indeed, the WNT/β-catenin pathway is known to critically regulate self-renewal and differentiation of neural stem/progenitor cells [23, 24]; moreover, it is essential for glioma tumorigenesis and for the maintenance of an undifferentiated state in GBM cells [9, 25]. We found through overexpression and silencing experiments that the higher β-catenin level in the stem-like cells of glioblastoma is dependent upon RYK; in addition, knock-down of β-catenin strongly suppressed the stemness phenotype of GBM cells. Finally, our rescue experiments demonstrate that transfection of β-catenin cDNA can partially recover the losses in sphere-forming ability and stemness-marker expression associated with RYK silencing.

In conclusion, we provide a novel function for RYK: it promotes the stemness of GBM cells, mediating this role through β-catenin signaling. In glioblastoma stem cells, RYK is overexpressed, is required for the activation of the pluripotency-related transcription-factor circuitry, and sustains neurosphere formation, leading to the establishment and maintenance of an undifferentiated state. As a consequence, blockade of RYK activation may represent a novel strategy for the treatment of GBM.

## MATERIAL AND METHODS

### Cell and neurosphere cultures

The glioblastoma cell line U87MG was obtained from American Type Culture Collection (ATTC, LG Standards, Milan, Italy); AM38 and U251MG cells were kindly donated by Dr Frank Furnari (La Jolla University, CA, USA). Cells were grown in Dulbecco's Modified Eagle's Medium (DMEM) supplemented with 10% heat-inactivated fetal bovine serum (FBS), 2 mM L-glutamine, and 100 U/ml penicillin/streptomycin (all media and supplements were purchased from Sigma-Aldrich, Milan, Italy, unless otherwise stated). For neurosphere cultures, single cells were plated at a density of 1,000 cells/ml and grown in serum-free DMEM-F12 supplemented with B27 (ThermoFisher Scientific, Milan, Italy), 10 ng/ml EGF, 20 ng/ml basic FGF (bFGF) (BD Biosciences, Milan, Italy), and antibiotic-antimycotics (ThermoFisher Scientific). After 5–7 days, neurospheres, which appeared as clusters of floating, viable cells, were collected by gentle centrifugation (800 rpm) and dissociated with 0.25% trypsin for 5 min.

### Glioblastoma stem-cell isolation and differentiation

GBM tissue specimens were obtained from adult patients undergoing craniotomy at the Institute of Neurosurgery, School of Medicine, Università Cattolica, Rome, Italy, as previously described [19, 26, 27]. Informed consent was obtained from patients before surgery. Stem cells were isolated through mechanical dissociation of GBM tumor tissue and cultured at low density in a serum-free medium supplemented with EGF and bFGF as described elsewhere [27]. To induce differentiation, cells were plated on flasks coated with BD Matrigel™ Basement Membrane Matrix (BD Biosciences) in the absence of EGF and bFGF for 2 weeks.

### Cell transfection

For transient RYK overexpression, cells were transfected with 4 μg of pcDNA3-h-*RYK* or pcDNA3. Ryk cDNA was kindly provided by Dr Stacker [28]. Lipopfectamin 2000 (Invitrogen, Thermo Fisher, Milan, Italy) was used for U87MG and patient-derived stem cells; X-tremeGENE9 DNA Transfection Reagent (Roche, Milan, Italy) was used for U251MG and AM38 cell lines.

To transiently knockdown *RYK* or β-catenin cDNA, 100nM of siRyk (Ambion, Thermo Fisher), siβ-catenin (Santa Cruz Biotechnology, MA, USA), or siRNA control (Ambion, Thermo Fisher) were transfected using Lipopfectamin 2000 for patient-derived stem cells or Oligofectamine (Invitrogen, Thermo Fisher) for all other cell lines.

### Protein isolation and Western blotting

Cells were washed twice in ice-cold PBS and then lysed in JS buffer (50 mM HEPES, pH 7.5, containing 150 mM NaCl, 1% glycerol, 1% Triton X-100, 1.5 mM MgCl_2_, 5 mM EGTA, 1 mM Na_3_VO_4_, and 1X protease inhibitor cocktail) as described [29]. Protein concentration was determined with a Bradford assay (Bio-Rad, Milan, Italy) using bovine serum albumin as standard; equal amounts of proteins were resolved on SDS-PAGE (10% acrylamide), electroblotted onto nitrocellulose membranes (G&E Healthcare, Milan, Italy), blocked for 1 h with 5% non-fat dry milk in Tris-buffered saline (TBS) containing 0.1% Tween-20, and incubated at 4°C overnight with primary antibody. Detection was performed by peroxidase-conjugated secondary antibodies (Santa Cruz Biotechnology) using enhanced chemiluminescence (EuroClone, Milan Italy). Primary antibodies were: anti-OCT3/4, anti-SOX2, anti-NANOG, anti-NESTIN (all from Santa Cruz Biotechnology), anti-EZH2, anti- β-catenin (from Cell Signaling Technology, EuroClone), anti-CD133 (Proteintech, Rosemont, IL, USA), anti-RYK (Genetex, CA, USA), and anti-β-actin (Sigma-Adrich).

### RNA extraction and real-time PCR

Total RNA was extracted using EuroGOLDTriFast (EuroClone, Milan, Italy) according to the manufacturer's protocol. Reverse transcription of total mRNA was performed as described [30] starting from equal amounts of total RNA/sample (500 ng) using SuperScript® III Reverse Transcriptase (Invitrogen, Milan, Italy). Quantitative analyses of *RYK*, *NANOG*, *OCT3/4*, *SOX2*, *EZH2*, nestin, *CD133*, β-catenin, and β-actin (as an internal reference) were performed by real-time PCR using specific primers (IDT, Bologna, Italy) and iQ^TM^ SYBR Green Supermix (Bio-Rad). All reactions were run in triplicate. To amplificate genes of interest we used the following primers:

β-ACTIN fw:5′- TGCGTGACATTAAGGAGAAG -3′, rv:5′-GCTCGTAGCTCTTCTCCA-3′;

NESTIN fw:5′-CAGGAGAAACAGGGCCTACA -3′, rv: 5′-AGCTGAGGGAAGTCTTGGAG-3′;

EZH2 fw:5′-GAGTTGGTGAATGCCCTTGG-3′, rv:5′-TGCTGTGCCCTTATCTGGAA-3′;

OCT3/4 fw:5′-CGAAAGAGAAAGCGAACCAG -3′, rv:5′-GCCGGTTACAGAACCACACT-3′;

SOX-2 fw:5′-GCACATGAACGGCTGGAGCAAC G-3′, rv:5′-GCTGCGAGTAGGACATGCTGTAGG-3′;

NANOG fw:5′-CAAAGGCAAACAACCCACTT -3′, rv:5′-TCTGGAACCAGGTCTTCACC-3′;

RYK fw:5′-TGTAAGCTGCGAGGTCTTCA-3′, rv:5′-TTGCTGAGAAATTGCCTGTG-3′;

β-CATENIN fw:5′-TCCCACTAATGTCCAGCGTT -3′; rv:5′-ATGGACCATAACTGCAGCCT-3′;

CD133 fw:5′-TTCTTGACCGACTGAGACCC-3′, rv:5′-CCAAGCACAGAGGGTXATTG-3′.

### Neurosphere-forming assay

Cells were plated in 60mm dishes in stem cell medium (see above). Colonies were counted under an inverted microscope (Nikon, Milan, Italy) and then photographed, as described.

### *In vitro* limiting dilution assay

Cells were seeded in stem cell medium (see above) at 1, 5 and 10 cells per well into a 96-well plate. One week after seeding, the number of wells containing spheroids for each cell plating density was counted, and Extreme limiting dilution analysis was performed using software available at http://bioinf.wehi.edu.au/software/elda

### Soft-agar assay

Cells were plated in 60mm dishes in a solution containing 2xDMEM, TPB buffer, and 1.25% noble agar (Difco, BDBiosciences), as previously described [31]. Briefly, cells were harvested, counted, and a 7-ml layer of noble agar solution left to polymerize on the bottom of the dishes. Cells were resuspended in 2ml of solution, seeded, and left to grow for 2 weeks in the incubator.

### Transwell migration assay

Dissociated tumor spheres were counted and 1.4 × 10^5^ cells/point were treated or transfected as indicated. Following 24 h, 1 × 10^5^ cells were seeded in the upper chamber of transwells (Corning, Corning, NY, USA) in serum-free DMEM and exposed to 10% FBS to induce migration (0.6 ml in the lower chamber) for an additional 24 h, as previously described [32]. Migrated cells were visualized by staining with 0.1% crystal violet in 25% methanol and photographed with Leica Application Suite. The percentage of migrated cells was evaluated by eluting crystal violet with 1% sodium dodecyl sulfate (SDS) and reading the absorbance at 594 nm wavelength.

### Statistical analysis

Continuous variables are given as mean ± 1 standard deviation. For comparison of two group, Student's *t*-test was used to determine differences between mean values for normal distribution. All data were analyzed for significance using GraphPad Prism 6 (San Diego, CA, USA) software, and a probability level < 0.05 was considered significant throughout.

## SUPPLEMENTARY MATERIALS


