# Supplementary material for: RYK promotes the stemness of glioblastoma cells via the WNT/β-catenin pathway
Source: Oncotarget. 2017 Jan 9;8(8):13476–87. doi: 10.18632/oncotarget.14564 (PMC5355113; doi:10.18632/oncotarget.14564)
Supplement: Supplementary file 1 [file oncotarget-08-13476-s001.pdf]

RYK promotes the stemness of glioblastoma cells via the WNT/β-catenin pathway

Supplementary Materials

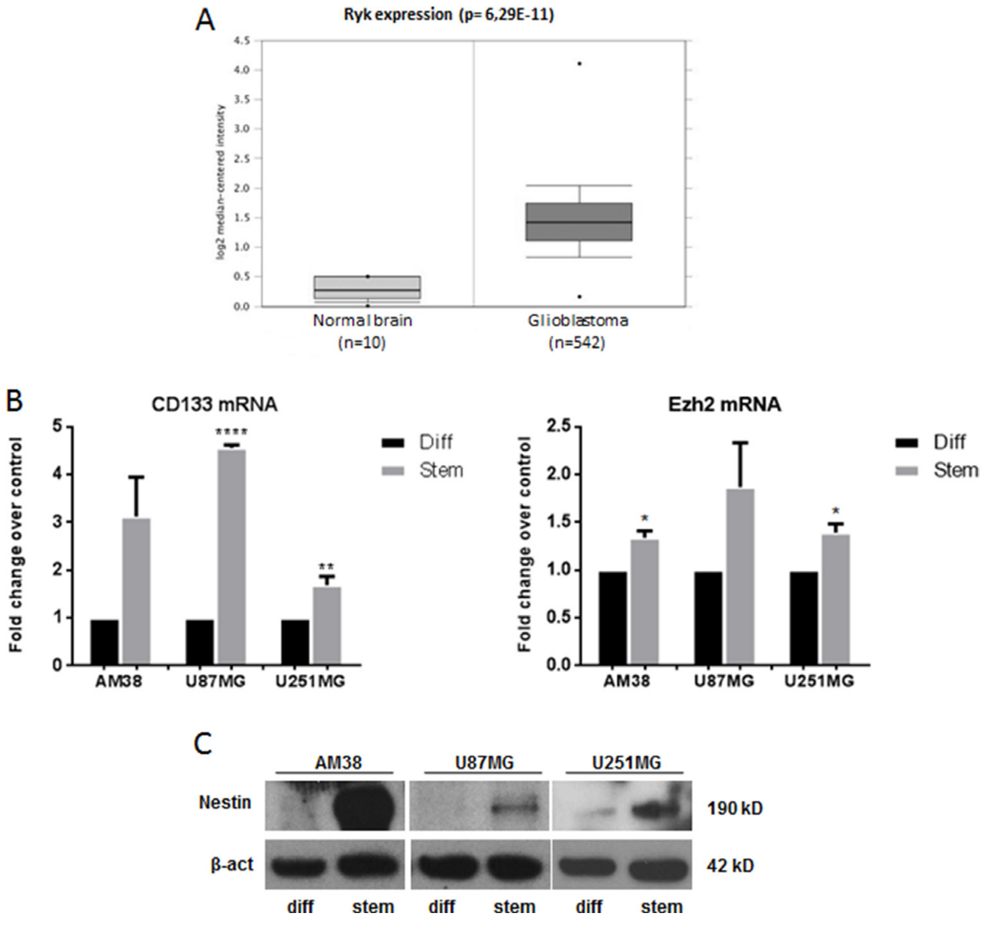

Supplementary Figure 1: Ryk expression in GBM samples and stemness markers in GSCs. (**A**) A significant increase in Ryk expression was identified in GBM tissues (n = 542) compared to normal brains (n = 10). Ryk expression data were obtained from Oncomine database. Real-time PCR (**B**) or Western blotting (**C**) were performed to analyze CD133, Ezh2 and Nestin mRNA or protein levels in GBM cell lines (AM38, U87MG and U251MG). Western blot analyses are from representative experiments, and β-actin was used as loading control. In c, the experiments were repeated at least two times. In b and c statistical significance was calculated using Student's *t*-test. $P < 0.05$ was considered significant, and the results are presented as mean ± SD. *$p < 0.05$; **$p < 0.01$; ****$p < 0.0001$. In (C) the blots representing Nestin for AM38 and U87MG are from the same gels of Figure 1F. Therefore they have the same β-actin normalization.

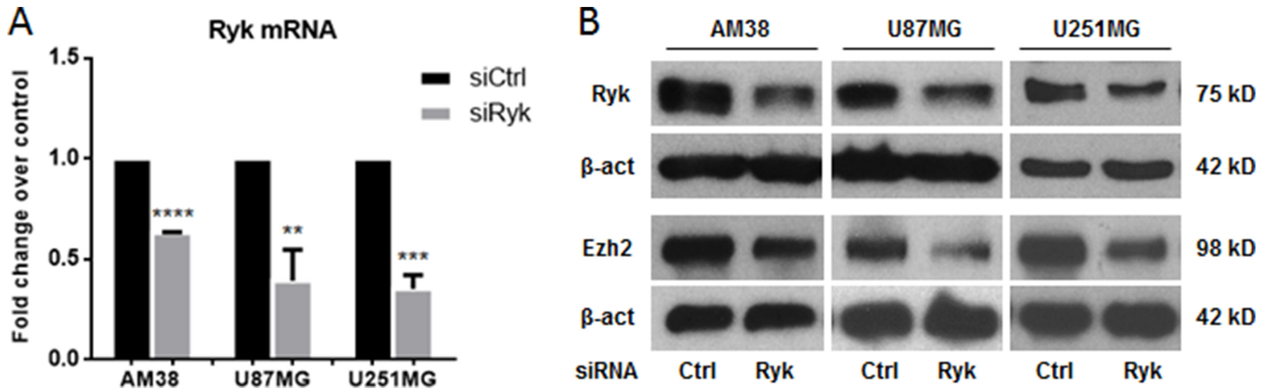

Supplementary Figure 2: siRyk transfection control and stemness marker expression. GBM cell lines (AM38, U87MG and U251MG) were transfected with Ryk siRNA or a control siRNA sequence. Real-time PCR (**A**) and/or Western blotting (**B**) were performed to analyze Ryk and Ezh2 mRNA and/or protein levels. Western blot analyses are from representative experiments, and β-actin was used as loading control. In (A) the experiments were repeated at least two times and statistical significance was calculated using Student's *t*-test. $P < 0.05$ was considered significant, and the results are presented as mean ± SD. $^{**}p < 0.01$; $^{***}p < 0.001$; $^{****}p < 0.0001$. In (B) the blot representing Ryk and Ezh2 for AM38 and U87MG are from the same gels of Figure 2F. Therefore they have the same β-actin normalization.

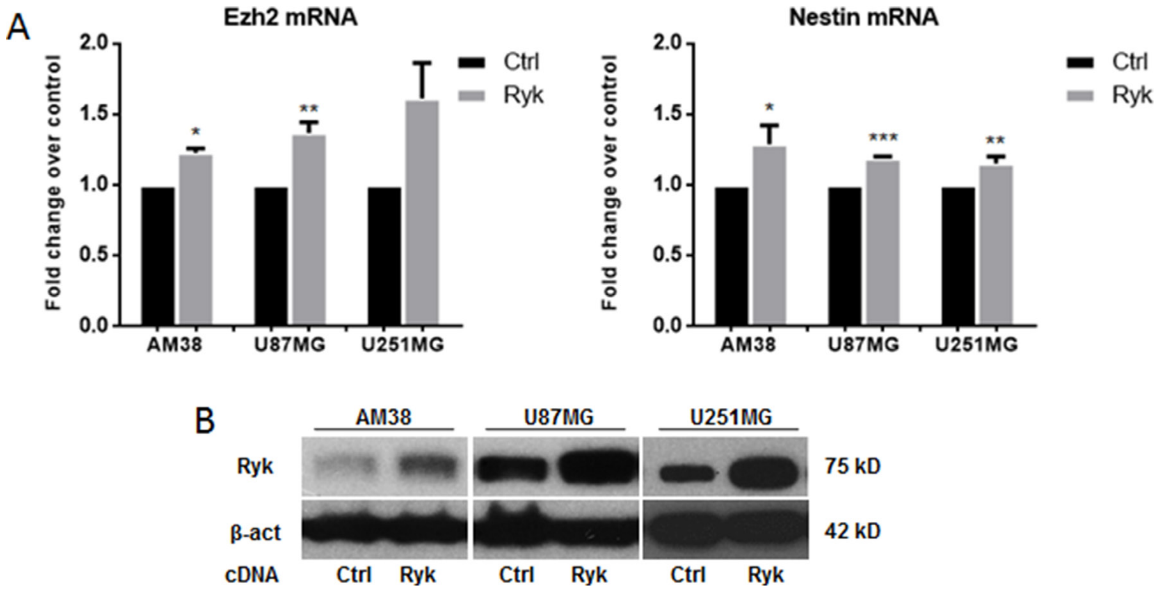

Supplementary Figure 3: Ryk transfection control and stemness marker expression. GBM cell lines (AM38, U87MG and U251MG) were transfected with Ryk cDNA or a control vector. Real-time PCR (**A**) or Western blotting (**B**) were performed to analyze Ryk, Ezh2 and Nestin mRNA or protein levels. Western blot analyses are from representative experiments, and β-actin was used as loading control. In (A) the experiments were repeated at least two times and statistical significance was calculated using Student's *t*-test. $P < 0.05$ was considered significant, and the results are presented as mean ± SD. $^{**}p < 0.01$; $^{***}p < 0.001$; $^{****}p < 0.0001$. In (B) the blot representing Ryk for AM38 is from the same gel of Figure 3C. Therefore they have the same β-actin normalization.
